# Transcriptomic Analysis Reveals a Sex-Dimorphic Influence of GAT-2 on Murine Liver Function

**DOI:** 10.3389/fnut.2021.751388

**Published:** 2021-09-16

**Authors:** Jian Fu, Qingzhuo Zhang, Zebiao Wu, Changming Hong, Congrui Zhu

**Affiliations:** ^1^State Key Laboratory for Conservation and Utilization of Subtropical Agro-Bioresources, Guangdong Laboratory of Lingnan Modern Agriculture, Guangdong Provincial Key Laboratory of Animal Nutrition Control, National Engineering Research Center for Breeding Swine Industry, College of Animal Science, South China Agricultural University, Guangzhou, China; ^2^Department of Diagnostic Medicine/Pathobiology, Kansas State University, Manhattan, KS, United States

**Keywords:** GAT-2, sex dimorphism, liver, amino acids, transcriptome

## Abstract

Accumulating evidence shows that the γ-amino butyric acid (GABA)ergic system affects the functions of different organs, and liver is one of the most sex-dimorphic organs in animals. However, whether and how the GABAergic system influences liver function in a sex-specific manner at the intrinsic molecular level remains elusive. In this study, firstly, we find that the levels of GABA are significantly increased in the livers of female mice with GABA transporter (GAT)-2 deficiency (KO) whereas it only slightly increased in male GAT-2 KO mice. Apart from the amino acid profiles, the expressions of toll-like receptors (TLRs) also differ in the livers of female and male KO mice. Moreover, RNA-seq results show 2,227 differentially expressed genes (DEGs) in which 1,030 are upregulated whereas 1,197 that are downregulated in the livers of female KO mice. Notably, oxidative phosphorylation, non-alcoholic fatty liver disease, Huntington's disease, and peroxisome proliferator-activated receptor (PPAR) signaling pathways are highly enriched by GAT-2 deficiency, indicating that these pathways probably meditate the effects of GAT-2 on female liver functions, on the other hand, only 1,233 DEGs, including 474 are upregulated and 759 are downregulated in the livers of male KO mice. Interestingly, retinol metabolism, PPAR signaling pathway, and tuberculosis pathways are substantially enriched by GAT-2 deficiency, suggesting that these pathways may be responsible for the effects of GAT-2 on male liver functions. Collectively, our results reveal the sex-dimorphic effects of GAT-2 in guiding liver functions, and we propose that targeting the GABAergic system (e.g., GATs) in a sex-specific manner could provide previously unidentified therapeutic opportunities for liver diseases.

## Introduction

The γ-aminobutyric acid transporter (GAT) family, which includes GAT-1, GAT-2, GAT-3, and BGT-1, is the important regulator in controlling the intracellular and extracellular γ-amino butyric acid (GABA) concentration (1, 2). GAT-1 or GAT-3 mainly expresses in the central nervous system and regulates the GABA activity in the central nervous system (3). On the contrary, GAT-2 is predominantly found in the peripheral organ, especially in liver and kidney, and it is considered as the GABA and taurine transporter in the liver (4). Except for the transport function, the physiological roles for GATs have also been described. For example, it has been demonstrated that GAT-2 could affect immune cell fates [e.g., macrophage polarization (5), Th1 differentiation (6), and Th17 responses (7)] and orchestrate the inflammatory responses of lipopolysaccharide-induced sepsis, infection-induced pneumonia, and high-fat diet-induced obesity *in vivo* (5, 8). Nevertheless, the physiological roles for GATs in directing the functions of peripheral organs are still enigmatic.

Liver is an organ mainly for metabolism and also plays a critical role in detoxification, biosynthesis of serum proteins, endocrine, and immune homeostasis (9, 10). More importantly, the liver is one of the most sexually dimorphic organs in gene expressions due to the different metabolic needs for male and female animals (11). For a long time, mice have been used as models for liver diseases such as non-alcoholic fatty liver disease (NAFLD), cirrhosis, fibrosis, and tumorigenesis (12–15). Also, studies have shown that there are significant gender differences in liver metabolism of male and female mice (16). Indeed, sex hormones (the conversion of testosterone to estradiol) meditate sexual dimorphism in the liver (11, 17). However, whether other endogenous signals modulate sexual dimorphism in the liver or influence liver functions in a sex-specific manner remains largely unclear.

In this study, the hepatic tissues of the wild type and GAT-2^−/−^ mice are performed by analyzing amino acids, quantitative reverse transcription PCR (RT-qPCR), and transcriptome sequencing. As the connection between GAT-2 gene and liver function is not fully elucidated, we hope to analyze the amino acid profile and differentially expressed genes that are related to inflammation, like toll-like receptors (TLRs) family, and provide a basis for researching the link between GAT-2 and liver function in different sexes.

## Materials and Methods

### Animal Administration

The GAT2^−/−^ mice (KO) have been described previously (7); the mice were housed in pathogen-free colonies (temperature, 20–30°C, relative humidity, 50–60%, 12 h dark/12 h light), and they had free choice of standard rodent feed and drinking water. The homozygous wild-type (WT) mice and KO mice were bred separately and serve as further studies.

### Sample Collection

Ten 9-week-old WT mice (half male and half female) and ten 9-week-old KO mice (half male and half female) were selected randomly in this study. The mice were sacrificed, and the livers were collected at necropsy. The samples were frozen in liquid nitrogen immediately and stored at −80°C for further analysis.

### Amino Acids Analysis

After accurately weighing the tissue samples (40.0 mg) in a centrifuge tube, 400 μl ultrapure water was added and then vortexed for 4.5 min. Then, 200 μl of homogenates was added to 800 μl of methanol–acetonitrile (50:50, V/V). The mixture was vortexed and sonicated in ice water at 4°C for 10 min and then centrifuged at 19,000 *g* for 15 min at 4°C. The supernatant was taken and vacuum-dried at 60°C for 90 min and then dried with nitrogen to obtain a dry substance. The dry substance was dissolved with 200 μl methanol–water (50:50, V/V) and sonicated in ice water at 4°C for 10 min before centrifugation at 19,000 *g* for 15 min at 4°C. The supernatant was filtered through a 0.22 μm membrane filter. The experiments were performed on a Thermo Fisher Scientific UPLC system (Dionex UltiMate 3000) coupled with a mass spectrometer (Q-Exactive Focus). Xcalibur software (version 3.0) was used for instrument control, data acquisition, and data analysis.

### RNA Extraction, Qualification, and Quantification

Total RNA of liver tissue was extracted using TRIZOL (Invitrogen, CA, USA) and then treated with DNase I (Invitrogen, CA, USA) according to the instruction of the manufacturer. The RNA quality was assessed by an RNA 6000 Nano Assay Kit and determined by Agilent Bioanalyser2100 (Agilent Technologies, CA, USA), and the RNA concentration was detected spectrophotometrically at 260 nm by the ND-2000 (NanoDrop Technologies, DE, USA).

### Quantitative Reverse Transcription PCR

Complementary DNA (cDNA) was reverse transcribed using the RevertAcidTMfrst strand cDNA synthesis kit (TaKaRa, Qingdao, China). The amplification reactions were performed using an ABI Prism 7900 HT sequence detection system (Applied Biosystems, Foster, CA, USA). Primers were designed using PrimerPremier 5.0 and β-actin was used as an internal control to normalize target gene transcript levels. The relative levels of genes were presented in terms of 2^−(ΔΔCt)^, where ΔCt = (Ct_target_ – Ct_β−actin_) _treatment_ – (Ct_target_−*Ct*_β−actin_)_control_.

### Library Construction and Sequencing

Total RNA was purified by using the RNA purification bead and fragmented into short fragments with 150–200 bp in length. Next, the short fragments were used as a template for synthesizing the strand cDNA using TruSeq Stranded mRNA LT Sample Prep Kit (Illumina, CA, USA) according to the instructions of the manufacturer, and the purification of strand cDNA was carried out using the Agencourt AMPureXP (Beckman Coulter, CA, USA). Then, the purified strand cDNA was carried out to the end reparation, “A” base addition, and sequencing adapt religation. Finally, cDNA was amplification and used to construct the library, which was sequenced on the Illumina sequencing platform (HiSeqTM 2500).

### Quality Control and Mapping

Raw data (raw reads) in fast q format were removed after ligation with NGS QC Tool kit software firstly, and then, the clean reads were obtained after removing the low-quality reads and reads containing ploy-N. The Q20, Q30, GC content, and sequence duplication level of the resultant clean sequences were calculated, and the high-quality clean reads were used for the further analysis.

We downloaded the reference genome and annotation files from the *Mus musculus* Genome Informatics (MGI, http://www.informatics.jax.org). We then used Bowtie2 (18) to build an index of the reference genome and TopHat (19) to align the paired-end clean reads with the reference genome.

### Analysis of DEGs, Cluster Analysis, GO, and KEGG Enrichment

The fragments per kilobase transcriptome per million mapped reads (FPKM) value was used to quantify transcripts expression, it was calculated using cufflinks, and the read counts of each gene were obtained by htseq-count. Differentially expressed genes (DEGs) were identified using the DESeq (2012) functions estimate Size Factors and nbinom Test. A *P* < 0.05 was set as the threshold for significantly differential expression. Hierarchical cluster analysis of DEGs was performed to explore the pattern of genes expression. Gene ontology (GO) enrichment and Kyoto Encyclopedia of Genes and Genomes (KEGG) pathway enrichment analysis of DEGs were, respectively, performed using R based on the hypergeometric distribution.

### Statistical Analyses

Data in this study were presented as means ± SEM and analyzed using Prism 6.0 (GraphPad Software, Inc., La Jolla, CA, USA). The data that conformed to normal distribution and had equal variance were analyzed by unpaired *t*-test between two groups; data that conformed to normal distribution and had unequal variance were analyzed by unpaired *t*-test with Welch's correction between two groups; data that did not conform to normal distribution were analyzed by non-parametric test between the two groups. Mean values were considered as statistically different at *P* < 0.05.

## Result

### The GAT-2 Differently Affects Amino Acid Profile of Livers in Male and Female Mice

**Figure 1:**
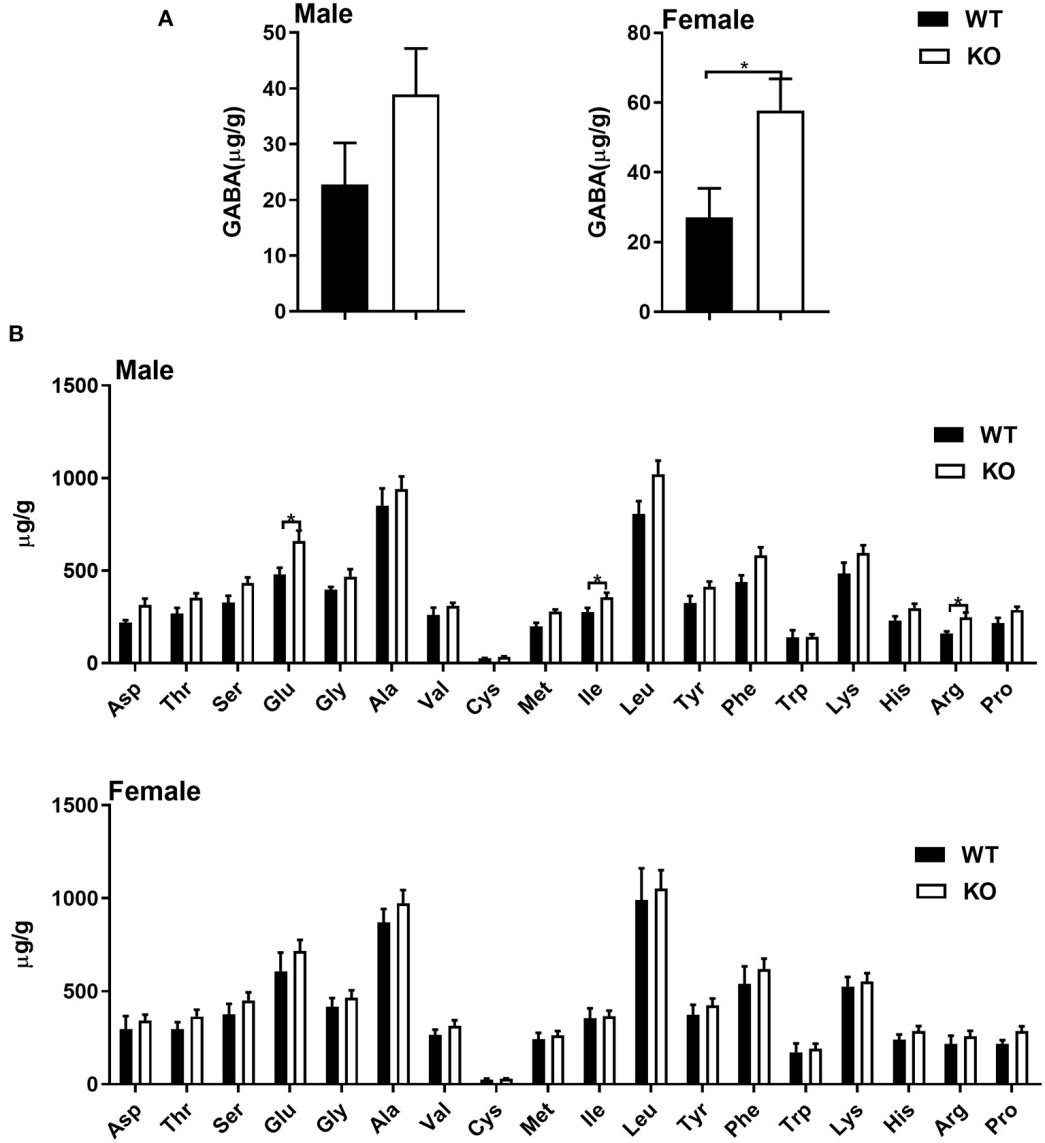
Common amino acids analysis by UPLC. **(A)** γ-amino butyric acid **(**GABA) level in livers of male (left) and female (right) mice. **(B)** Common amino acid level in livers of male (up) and female (down) mice. Data were analyzed with unpaired *t*-test and represented as means ± SEM except indicated.

Considering GAT-2 is the transporter for GABA, and liver is a crucial metabolically active organ, we analyzed the amino acid profile of livers in male and/or female mice with or without GAT-2 deficiency. In the livers of male KO mice, seven amino acids (glutamate, sarcosine, cystathionine, isoleucine, 5-hydroxylysine, 3-methylhistidine, and arginine) increased, but three amino acids (taurine, urea, and b-alanine) decreased (Figures 1A,B; Supplementary Figure 1A). Compared with WT mice, only two amino acids (sarcosine and GABA) increased in the livers of female KO mice (Figures 1A,B; Supplementary Figure 1B). The levels of GABA significantly increased in the livers of female KO mice, whereas it only slightly increased in male KO mice. These findings suggest that GAT-2 influences amino acid metabolism in liver of male mice and female mice in a different manner.

### The GAT-2 Differently Influences Expression of TLRs in Livers of Male and Female Mice

**Figure 2:**
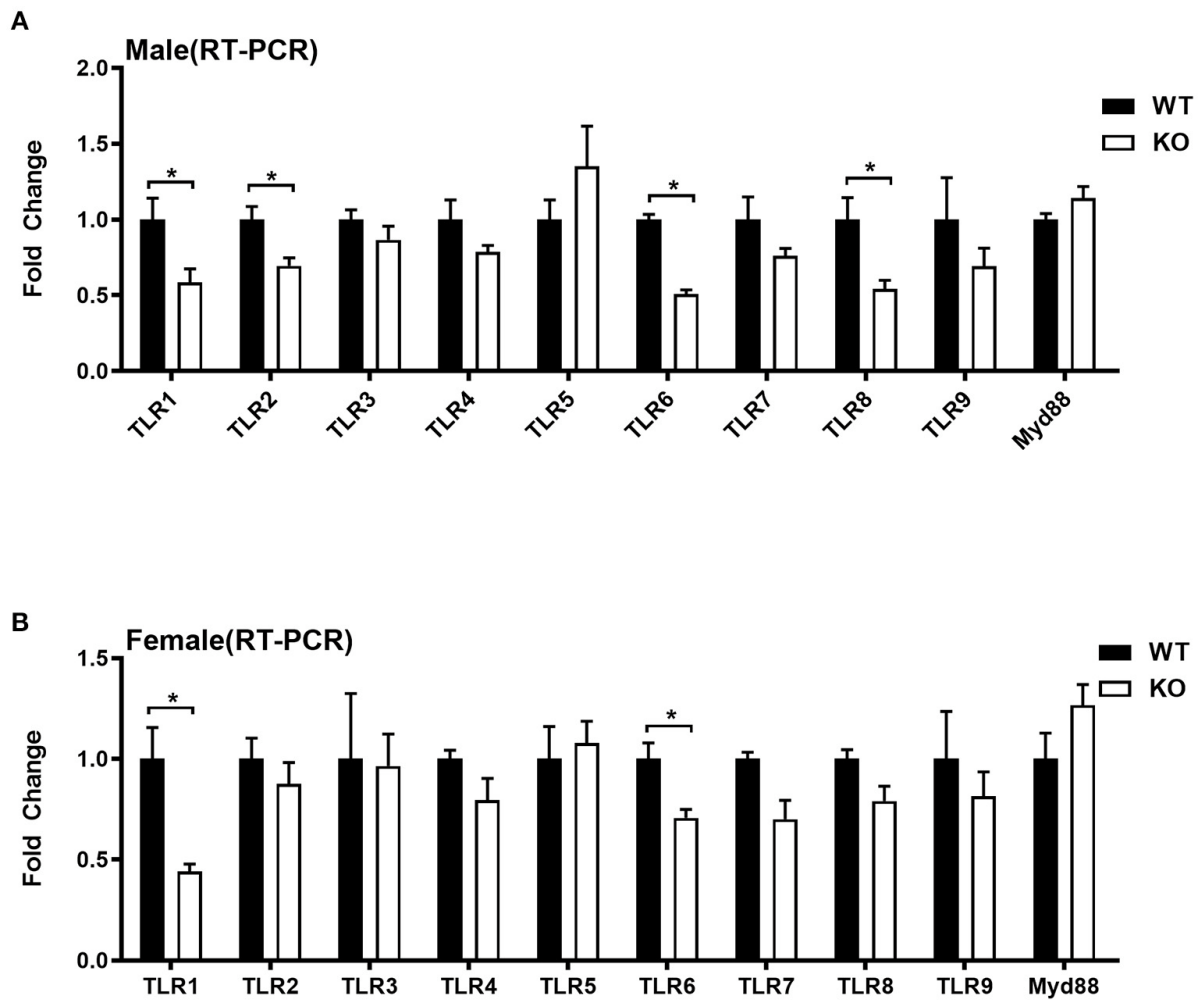
Quantitative reverse transcription PCR (qRT-PCR) validation expression profile of genes related to toll-like receptors (TLRs). [**(A)** male, **(B)** female]. Ten individual genes involved in toll-like receptor signaling pathways were analyzed by qRT-PCR. Data were analyzed with unpaired *t*-test and represented as means ± SEM except indicated. *P < 0.05.

Given that liver also serves as an important immune organ, we investigated whether GAT-2 affects the immunologic functions of livers in male and/or female mice. Here, we selected 10 genes, such as TLR1, TLR2, TLR3, TLR4, TLR5, TLR6, TLR7, TLR8, TLR9, and Myd88, which are related to TLR signaling pathways. RT-qPCR analysis revealed that TLR1, TLR2, TLR6, and TLR8 were downregulated in the livers of male KO mice (Figure 2A), whereas only TLR1 and TLR6 were downregulated in the livers of female KO mice (Figure 2B). Similarly, these findings indicate that GAT-2 may have a broader effect on TLR signaling of liver in male mice than those in female mice.

### Sequencing and Transcriptome Assembly

Subsequently, we determined the sex-specific influence of GAT-2 on liver function at the intrinsic molecular level by performing the RNA-seq method. After Illumina sequencing, the sequencing and assembly are displayed in Table 1. Totally, we achieved 60,629,043 and 59,038,390 raw reads in WT female and male mice libraries, and 58,760,836 and 60,090,923 raw reads in KO female and male mice libraries, respectively. After quality control, we retained 59,052,921 and 57,646,065 clean reads in WT female and male mice libraries, and 57,398,020 and 58,618,110 in KO female and male mice libraries, respectively. The GC content of WT female and male mice was 48.00 and 47.33%, respectively. The GC content of KO female and male mice was 48.17 and 47.50%, respectively.

**Table 1:** Principal features of sequencing reads mapping to the reference genome.

**Sample names**	**WT**		**KO**	
	**Female**	**Male**	**Female**	**Male**
Raw reads	60,629,043	59,038,390	58,760,836	60,090,923
Clean reads	59,052,921	57,646,065	57,398,020	58,618,110
Clean bases	7,378,808,677	7,203,159,866	7,172,154,188	7,324,579,947
Q30 (%)	95.11%	95.38%	95.41%	95.28%
GC content (%)	48.00%	47.33%	48.17%	47.50%
Total mapped	54,368,696 (92.07%)	47,232,185 (81.92%)	51,999,399 (90.61%)	48,125,748(82.13%)
Uniquely mapped	47,659,080 (80.69%)	39,741,345 (68.94%)	45,640,794 (79.54%)	40,733,721 (69.52%)
Reads map to ‘+’	23,787,334 (40.27%)	19,926,284 (34.56%)	22,801,521 (39.73%)	20,403,610 (34.82%)
Reads map to ‘–’	23,871,746 (40.42%)	19,815,061 (34.37%)	22,839,273 (39.80%)	20,330,111 (34.70%)
Non-splice reads	26,649,459 (45.13%)	23,731,299 (41.16%)	25,919,869 (45.19%)	23,817,410 (40.64%)
Splice reads	21,009,622 (35.56%)	16,010,046 (27.77%)	19,720,924 (34.35%)	16,916,311 (28.88%)

‘*+’ refers to sense strands; ‘–’ refers to anti-sense strands. ‘Non-splice reads’ refers to reads for the entire sequence is mapped to one exon; ‘Splice reads’, also called ‘junction reads’, refers to reads mapped to the border of exon*.

### Mapping Reads to the Transcriptome

We then mapped the clean reads to the *Mus musculus* Genome Informatics (MGI, http://www.informatics.jax.org), and the results showed that approximately 92.07 and 81.92% of clean reads in WT female and male mice, respectively, were matched with the reference genome, and approximately 90.61 and 82.13% of clean reads in KO female and male mice, respectively, were matched with the reference genome. Furthermore, 80.69 and 68.94% clean reads in WT female and male mice, respectively, and 79.54 and 69.52% clean reads in KO female and male mice, respectively, were uniquely mapped to the reference genome. However, more than 40% of the clean reads were non-splice reads in each library (Table 1).

### Annotation

**Figure 3:**
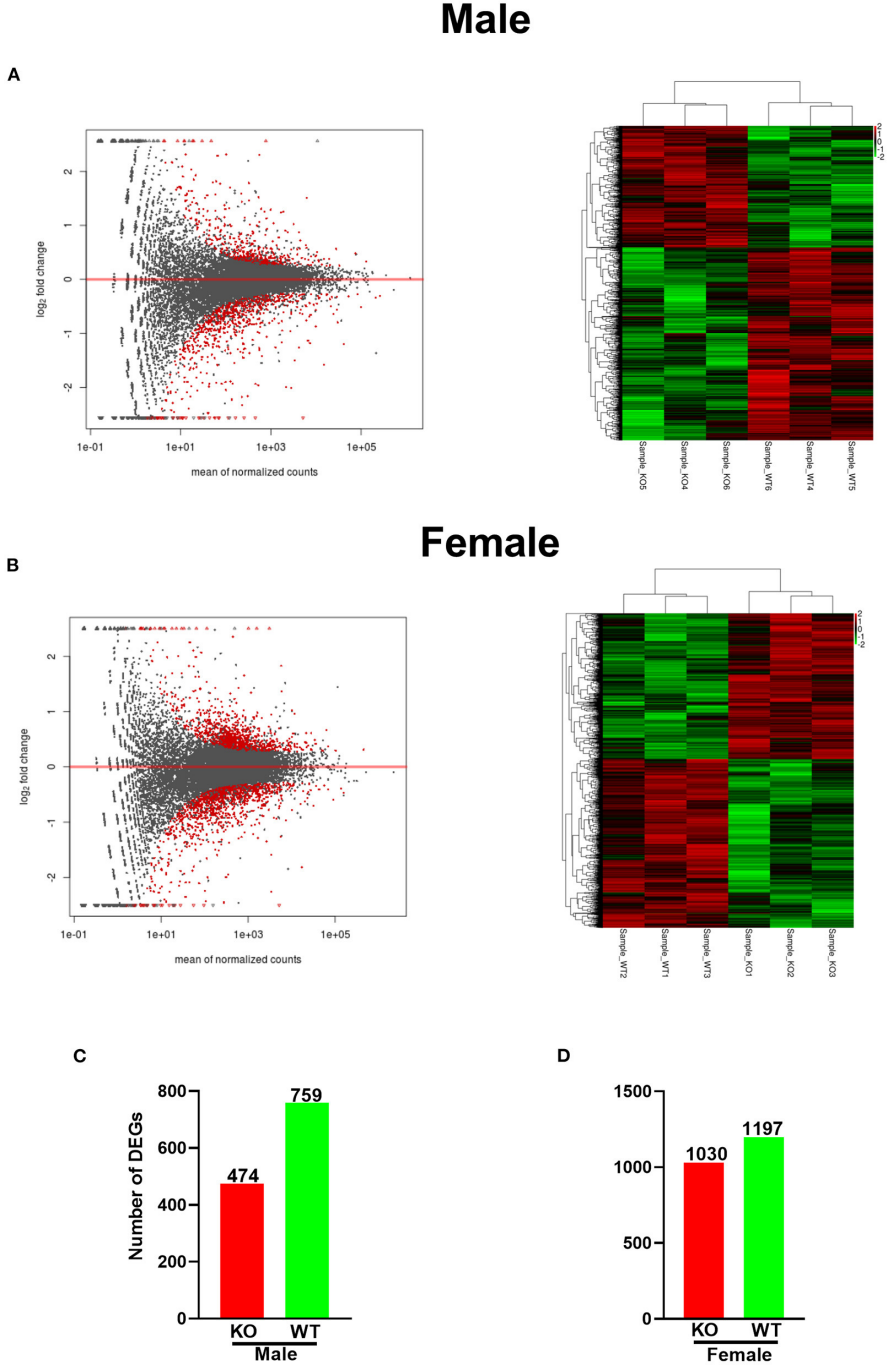
GABA transporter 2_(GAT2) deficiency alters the transcriptomic profile in the livers of male and female KO mice. **(A)** MA plot and heatmap analysis of upregulated (red) or downregulated genes (green) in male KO and wild-type (WT) livers (*n* = 3). **(B)** MA figure and heatmap analysis of upregulated (red) or downregulated genes (green) in female KO and WT livers (*n* = 3). **(C,D)** [**(C)**, male; **(D)**, female]. The number of up-/downregulated differentially expressed genes (DEGs) in female and male KO livers compared with WT livers, respectively. The red bars represented genes that were upregulated in KO compared to WT, while the green bars represented genes that were downregulated.

Both fold change and *p*-value or false discovery rate (FDR) were chosen to compare and analyze the differential expression of the Unigenes in two groups, and we screened differently expressed genes between the two groups based on the criteria |log2(fold change) |>0.0 and *P* < 0.05. The MA figures and heatmaps are shown in Figures 3A,B. Totally, 2,227 DEGs were identified in female KO mice, in which 1,030 genes were upregulated and 1,197 genes were downregulated (Figure 3C). However, only 1,233 DEGs were identified in male KO mice, in which 474 genes were upregulated and 759 genes were downregulated (Figure 3D). Interestingly, our RNA-seq data also demonstrated that GAT-2 may have a broader effect on TLR signaling of liver in male mice than those in female mice, evidenced by TLR1, TLR2, TLR3, TLR4, TLR6, and TLR8 that were downregulated in male KO mice (Supplementary Figure 2A), whereas only TLR1, TLR3, and TLR5 were downregulated and Myd88 were upregulated in KO female mice (Supplementary Figure 2B).

**Figure 4:**
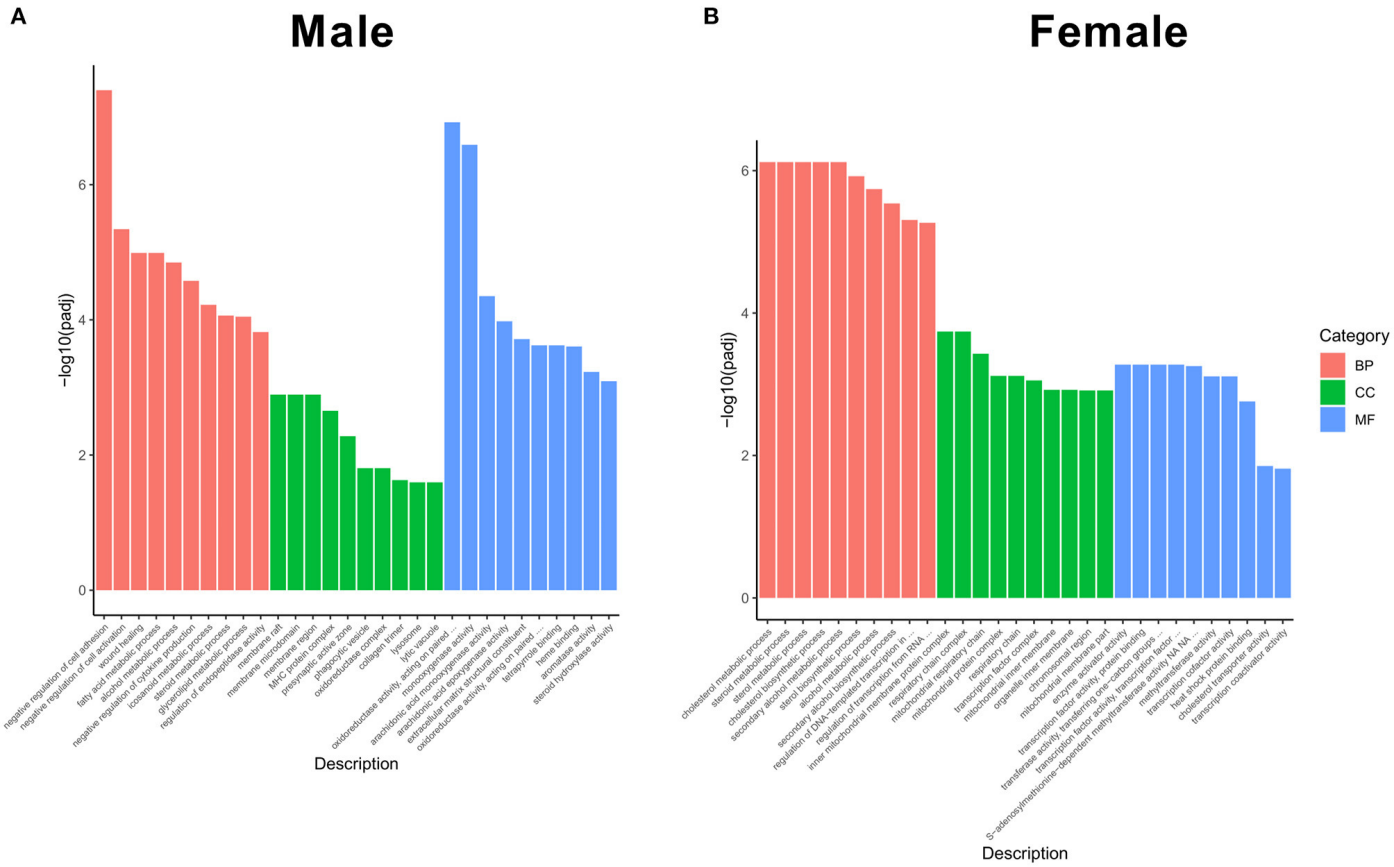
Functional GO categories of DEGs (KO vs WT). **(A)** GO classification in male KO and WT livers, including biological process, cellular component, and molecular function. **(B)** GO classification in female KO and WT livers, including biological process, cellular component, and molecular function. *Y*-axis: The logarithmic value of *P*-value, *X*-axis: GO classification.

After achieving the DEGs, we performed the GO enrichment analysis and functional annotation of DEGs. In the GO analysis, gene functions were divided into three categories, namely, biological process (BP), cellular component (CC), and molecular function (MF). The threshold value for significant enrichment of a GO term was *P* < 0.05. The top 10 differences in the significance of GO terms in BP, CC, and MF are shown in Figure 4. In the biological process, negative regulation of cell adhesion and cell activation was the most significant categories in male KO mice (Figure 4A, left), whereas cholesterol metabolic process and steroid metabolic process were the most significant categories in female KO mice (Figure 4B, left), and as for cellular component, membrane raft and membrane microdomain were the most significant in male KO mice (Figure 4A, middle), while the most significant categories were inner mitochondrial membrane protein complex and respiratory chain complex in female KO mice (Figure 4B, middle); in molecular function, oxidoreductase activity and monooxygenase activity were the most significant GO terms in male KO mice (Figure 4A, right), while enzyme activator activity and transcription factor activity were the most significant GO terms in KO female mice (Figure 4B, right).

**Figure 5:**
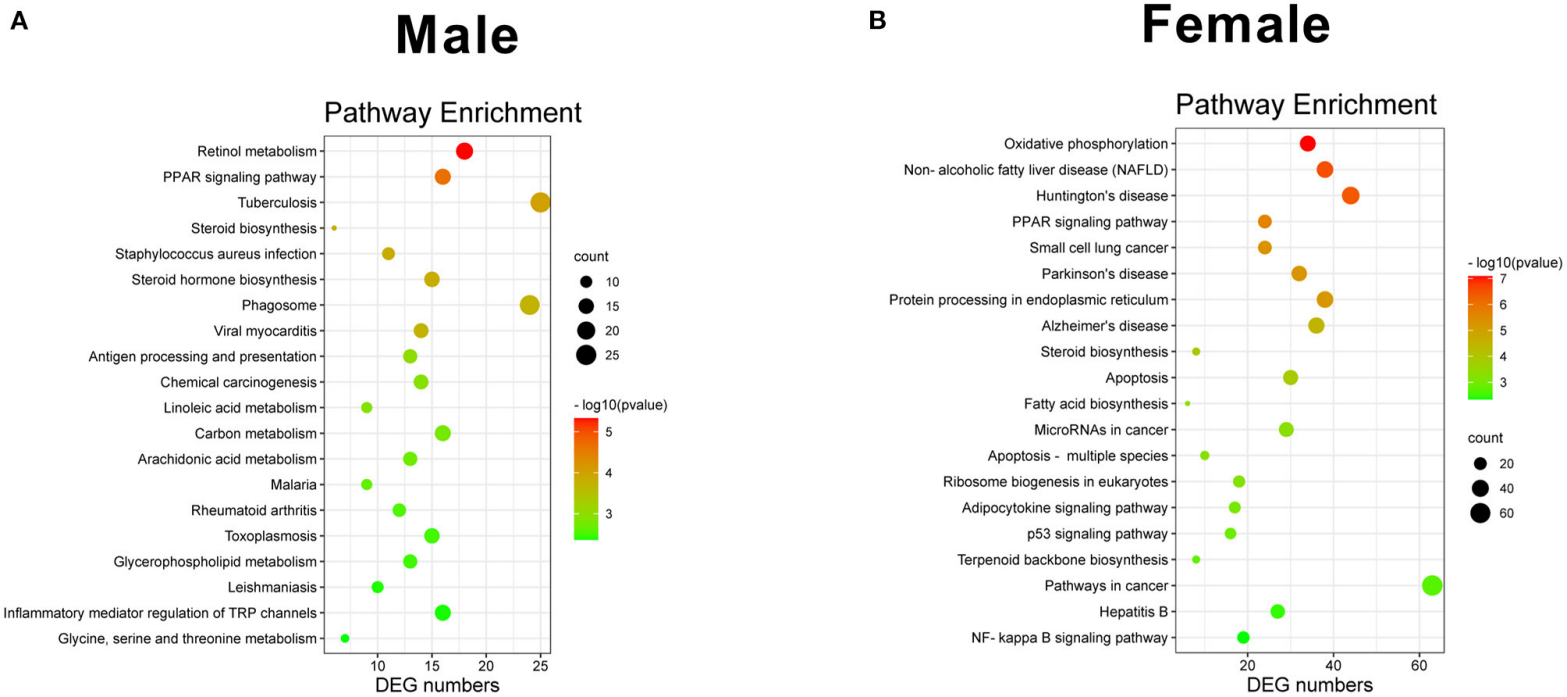
Scatterplot of enriched KEGG pathways for DEGs. [**(A)** male, **(B)** female]. Sizes of bubbles indicate the number of DEGs in the corresponding pathway, and the colors of the bubble represent *P*-value after multiple hypothesis test.

Kyoto Encyclopedia of Genes and Genomes was the main public database for pathways (20). In total, 284 and 277 different pathways were detected in KO female and male mice, respectively. The most significant 20 pathways are shown in Figure 5. Among these pathways, retinol metabolism, PPAR signaling pathway, and tuberculosis were the most three significant pathways in male KO mice (Figure 5A), whereas oxidative phosphorylation, NAFLD, and Huntington's disease were the most three significant pathways in KO female mice (Figure 5B). Interestingly, the PPAR signaling pathway showed a similar *p*-value in both male and female KO mice pathway enrichment analysis. Overall, our RNA-seq data suggest that GAT-2 influences liver function in a sex-specific manner at the intrinsic molecular level.

## Discussion

γ-amino butyric acid (GABA)ergic system consists of GABA, GABA receptors, glutamate decarboxylase (GAD), vesicular inhibitory amino acid transporter (VIAAT), GATs, and GABA transaminase (GABA-T). Studies show that the GABAergic system not only has inhibitory functions in the central nervous system but also plays vital roles in modulating the immune system. For example, GABA inhibits the development of proinflammatory T-cell responses (21). Besides the development of immune cells, GABA also modulates the release of inflammatory cytokines from peripheral blood mononuclear cells and T cells (22). GAD, and GABA receptors and transporters have been found in immune cells (e.g., macrophages and T cells) and could determine the fates of these immune cells (5, 7). Thus, these findings draw much attention in exploring the role of the GABAergic system in regulating the peripheral system and organs (including liver).

Liver is one of the most sexual-dimorphism organs and mainly for metabolism (23–25). Firstly, we analyzed the levels of GABA and other amino acids in murine livers. Interestingly, we demonstrate that GABA significantly increases in livers of KO female mice whereas slightly increases in the livers of male KO mice, indicating that GAT-2 deletion seems to affect the transport of GABA in livers of mice. Additionally, sarcosine increases in livers of both female and male KO mice, which could function as a potentially important metabolic intermediary of cancer cell invasion and aggressivity (26, 27). Isoleucine can improve the function of the immune system, including immune organs, cells, and reactive substances (28), whereas higher levels of glutamate, 5-hydroxylysine, and 3-methylhistidine may cause adverse health effects (29–31). Considering that GAT-2 deficiency mainly increases these aforementioned amino acids in the livers of male mice, it could be concluded that GAT-2 has a different immunomodulatory role in regulating the hepatic functions of male mice. However, the potential mechanisms still need to be further revealed.

Toll-like receptors are a group of important proteins involved in innate immunity and also serve as a bridge connecting non-specific immunity and specific immunity (32). They could recognize moderated microbial structures, such as bacterial lipopolysaccharide and viral double-stranded RNA, and activate signaling pathways that lead to immune responses against microbial infections (33). All TLRs share a traditional adaptor protein-MyD88 and also activate a common signaling pathway that culminates in the activation of nuclear factor–κB (NF-κB) transcription factors and the mitogen-activated protein kinases (MAPKs) extracellular signal (34, 35). Therefore, the expressions of TLR1, TLR2, TLR3, TLR4, TLR5, TLR6, TLR7, TLR8, TLR9, and Myd88 that are related to TLR signaling pathways are taken as the proxy for exploring the immunomodulatory role of GAT-2 on the hepatic functions. Intriguingly, both results from RT-qPCR and RNA-seq highlight that GAT-2 deficiency significantly lowers the expressions of TLR1/2/6/8 in male murine livers whereas it only highly downregulates TLR1 in the livers of female mice.

To further investigate the effects of GAT-2 on the livers at the molecular level, we perform the transcriptome analysis between the liver of WT mice and GAT-2^−/−^mice. After quality control, we have retained 59,052,921 and 57,646,065 clean reads in WT female and male mice libraries, 57,398,020 and 58,618,110 in KO female and male mice libraries, respectively, and the GC content is between 40 and 60%. These data mean that the sequence coverage of two libraries is enough for transcriptional analysis. Notably, we find that the DEG number of female KO mice is much larger in male KO mice (2,227 vs. 1,233), which indicates that the broader effects of GAT-2 on transcriptomic profile in the livers of female mice.

Differentially expressed genes are involved in a wide variety and different functions including biological process, cellular component, and molecular function (36). We can seek their differences more specifically in this study from KEGG analysis. Moreover, KEGG analysis shows that oxidative phosphorylation (OXPHOS) and NAFLD are the most significant pathways in KO female mice, whereas retinol metabolism and the PPAR signaling pathway are the most significant pathways in KO male mice. These findings indicate that GAT-2 might influence the metabolism of female and male liver functions through different pathways. OXPHOS is one of the dominant metabolic routes mainly for adenosine triphosphate (ATP) production in the cell (5, 37). NAFLD is defined as the excessive deposition of fat in hepatocytes, excluding alcohol and other specific factors that may damage the liver (13). Moreover, it has been shown that retinol promotes normal growth and development of the host (38). PPAR signaling pathway regulates the expression of genes associated with lipid metabolism, fat formation, maintenance of metabolic homeostasis, and inflammation (39). Therefore, GAT-2 might regulate excessive fat deposition and inflammation in the livers of female mice, whereas GAT-2 could affect normal growth and development of livers in male mice involving retinol metabolism and PPAR signaling pathway. Interestingly, the PPAR signaling pathway is significantly enriched in both male and female GAT-2 KO mice, which might attribute to the increase of GABA and/or affected TLRs. Nevertheless, well-designed experiments for validating the aforementioned results are needed.

## Conclusions

Collectively, our results reveal a sex-dimorphic influence of GAT-2 on murine liver functions at the molecular level, which could provide a basis for further discussing the physiological and pathophysiological role of GAT-2.

## Data Availability Statement

The datasets presented in this study can be found in online repositories. The names of the repository/repositories and accession number(s) can be found in the article/Supplementary Material.

## Ethics Statement

The animal study was reviewed and approved by Laboratory Animal Ethical Commission of the South China Agricultural University.

## Author Contributions

JF and CZ designed the research and analyzed data. JF, QZ, ZW, and CH performed the experiments and analyzed the data. JF wrote the manuscript. CZ revised the manuscript. All authors contributed to the article and approved the submitted version.

## Funding

This work was supported by the Guangdong Basic and Applied Basic Research Foundation (2019B1515210002).

## Conflict of Interest

The authors declare that the research was conducted in the absence of any commercial or financial relationships that could be construed as a potential conflict of interest.

## Publisher's Note

All claims expressed in this article are solely those of the authors and do not necessarily represent those of their affiliated organizations, or those of the publisher, the editors and the reviewers. Any product that may be evaluated in this article, or claim that may be made by its manufacturer, is not guaranteed or endorsed by the publisher.

## Supplementary Material

Supplementary Figure 1 Uncommon amino acids analysis by UPLC. [**(A)** male, **(B)** female]. Data were analyzed with unpaired *t*-test and represented as means ± SEM except indicated. ^*^*P* < 0.05.

Supplementary Figure 2 RNA-seq validation expression profile of genes related to TLRs. [**(A)** male, **(B)** female]. ^*^*P* < 0.05, ^**^*P* < 0.01, ^***^*P* < 0.001, ^****^*P* < 0.0001.

Supplementary Table 1 The full list of DEGs.

Supplementary Table 2 The full list of enriched GO terms.

Supplementary Table 3 Detailed information on the enriched KEGG pathways.
