# Supplementary figures and images for: Transcriptomic Analysis Reveals a Sex-Dimorphic Influence of GAT-2 on Murine Liver Function
Source: Front Nutr. 2021 Sep 16;8:751388. doi: 10.3389/fnut.2021.751388 (PMC8481587; doi:10.3389/fnut.2021.751388)

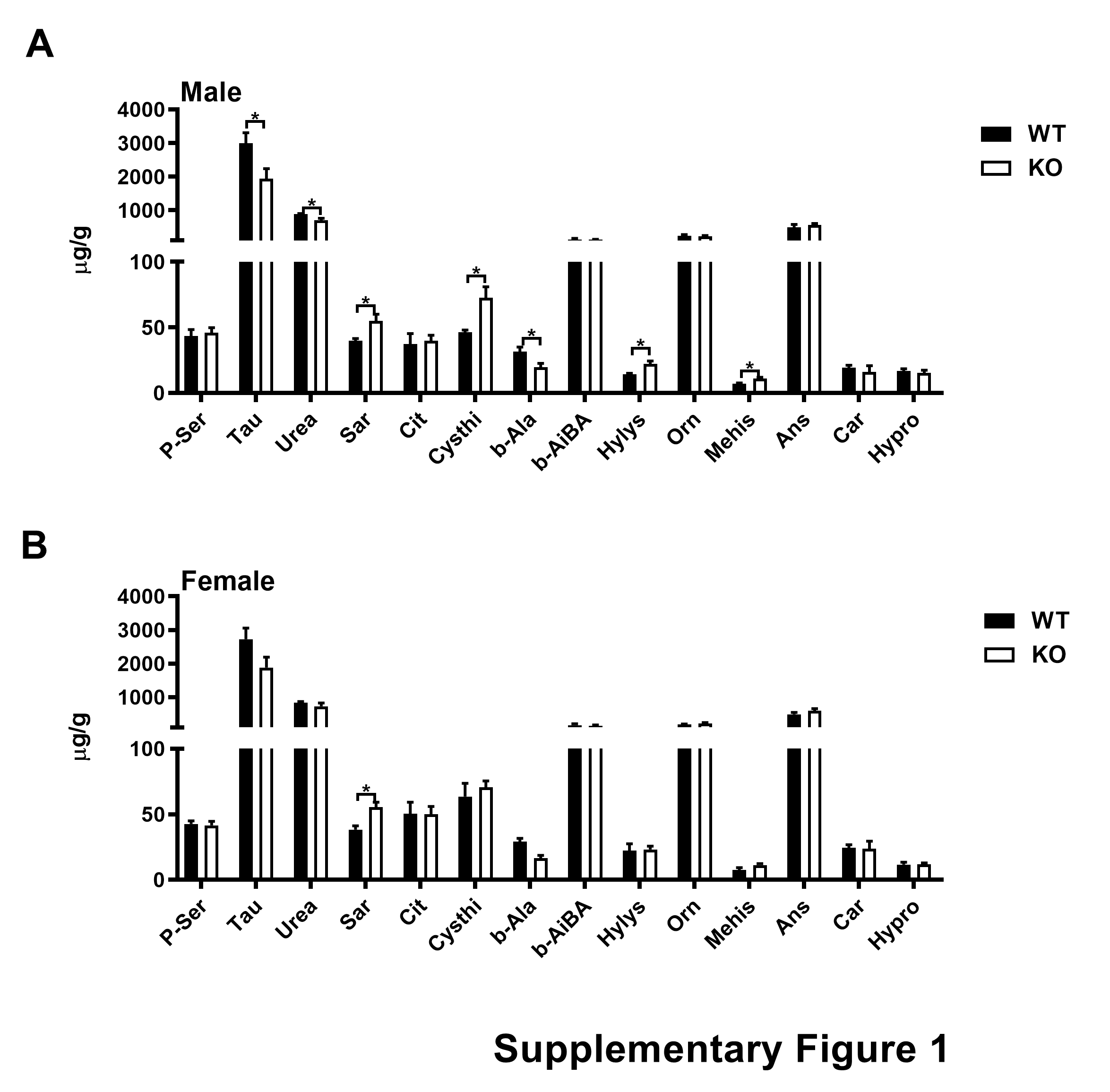

Supplement: Supplementary Figure 1 — Uncommon amino acids analysis by UPLC. [(A) male, (B) female]. Data were analyzed with unpaired t-test and represented as means ± SEM except indicated. *P < 0.05. [file Image_1.TIF]

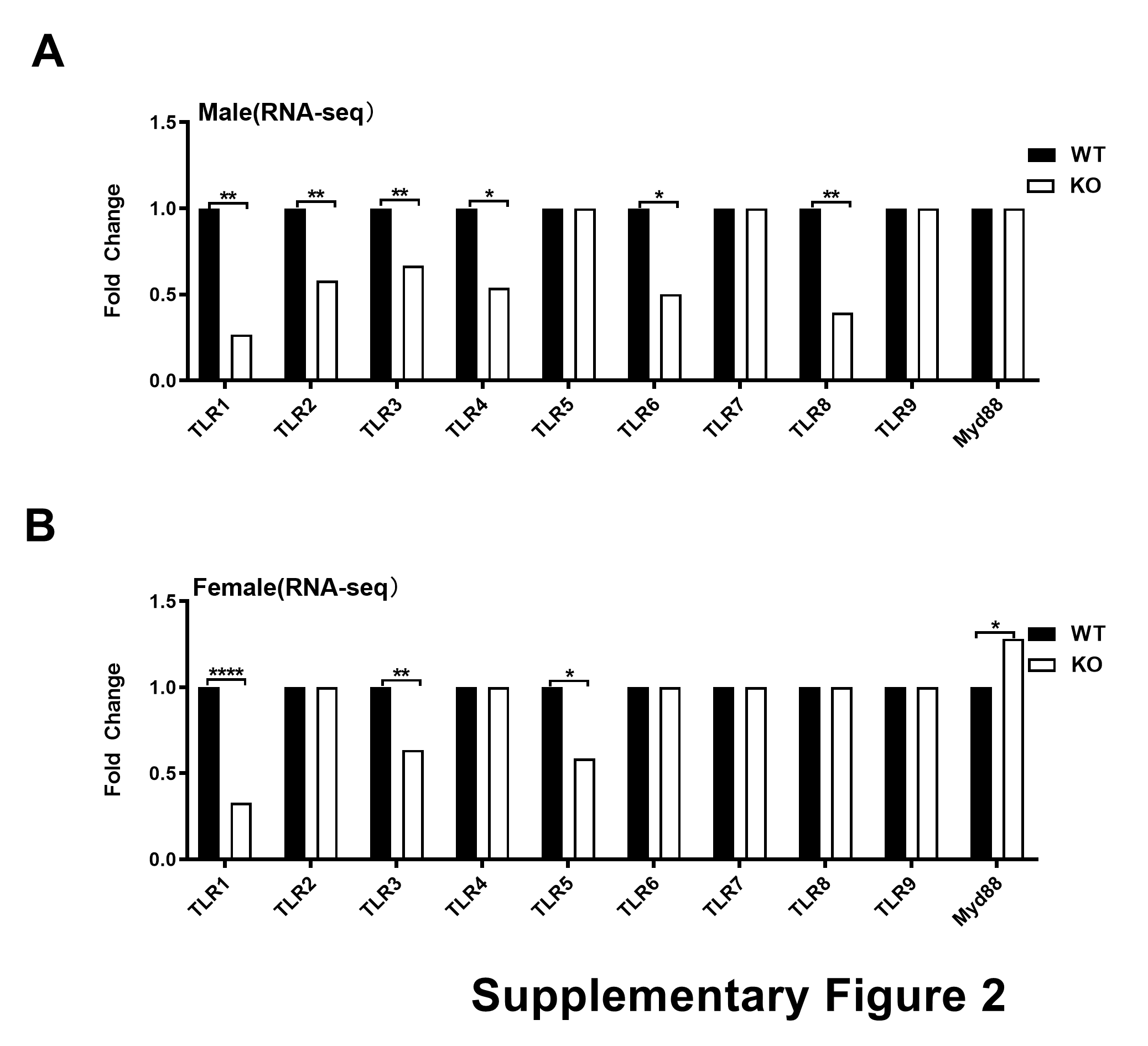

Supplement: Supplementary Figure 2 — RNA-seq validation expression profile of genes related to TLRs. [(A) male, (B) female]. *P < 0.05, **P < 0.01, ***P < 0.001, ****P < 0.0001. [file Image_2.TIF]
